# Restricting Microbial Exposure in Early Life Negates the Immune Benefits Associated with Gut Colonization in Environments of High Microbial Diversity

**DOI:** 10.1371/journal.pone.0028279

**Published:** 2011-12-22

**Authors:** Imke E. Mulder, Bettina Schmidt, Marie Lewis, Margaret Delday, Christopher R. Stokes, Mick Bailey, Rustam I. Aminov, Bhupinder P. Gill, John R. Pluske, Claus-Dieter Mayer, Denise Kelly

**Affiliations:** 1 Gut Immunology Group, University of Aberdeen, Rowett Institute of Nutrition and Health, Aberdeen, United Kingdom; 2 Veterinary Pathology, Infection & Immunity, School of Clinical Veterinary Science, University of Bristol, Bristol, United Kingdom; 3 Agricultural and Horticultural Development Board, Milton Keynes, United Kingdom; 4 School of Veterinary and Biomedical Sciences, Murdoch University, Murdoch, Western Australia, Australia; 5 Biomathematics & Statistics Scotland, University of Aberdeen, Rowett Institute of Nutrition and Health, Aberdeen, United Kingdom; Charité, Campus Benjamin Franklin, Germany

## Abstract

**Background:**

Acquisition of the intestinal microbiota in early life corresponds with the development of the mucosal immune system. Recent work on caesarean-delivered infants revealed that early microbial composition is influenced by birthing method and environment. Furthermore, we have confirmed that early-life environment strongly influences both the adult gut microbiota and development of the gut immune system. Here, we address the impact of limiting microbial exposure after initial colonization on the development of adult gut immunity.

**Methodology/Principal Findings:**

Piglets were born in indoor or outdoor rearing units, allowing natural colonization in the immediate period after birth, prior to transfer to high-health status isolators. Strikingly, gut closure and morphological development were strongly affected by isolator-rearing, independent of indoor or outdoor origins of piglets. Isolator-reared animals showed extensive vacuolation and disorganization of the gut epithelium, inferring that normal gut closure requires maturation factors present in maternal milk. Although morphological maturation and gut closure were delayed in isolator-reared animals, these hard-wired events occurred later in development. Type I IFN, IL-22, IL-23 and Th17 pathways were increased in indoor-isolator compared to outdoor-isolator animals during early life, indicating greater immune activation in pigs originating from indoor environments reflecting differences in the early microbiota. This difference was less apparent later in development due to enhanced immune activation and convergence of the microbiota in all isolator-reared animals. This correlated with elevation of Type I IFN pathways in both groups, although T cell pathways were still more affected in indoor-reared animals.

**Conclusions/Significance:**

Environmental factors, in particular microbial exposure, influence expression of a large number of immune-related genes. However, the homeostatic effects of microbial colonization in outdoor environments require sustained microbial exposure throughout development. Gut development in high-hygiene environments negatively impacts on normal succession of the gut microbiota and promotes innate immune activation which may impair immune homeostasis.

## Introduction

The mammalian intestine is colonized immediately after birth by commensal bacteria derived from the maternal vagina, faeces and skin as well as the external environment [1]. Thereafter, the composition of the gut microbiota is characterized by fluctuating changes in microbial diversity during the first years of life until an eventual convergence towards an adult microbiota [2]–[6]. This early-life succession and stabilization of the gut microbiota occurs concomitantly with the development and functional expansion of the mucosal immune system. Immune maturation is directly influenced by the presence of commensal bacteria [7]–[10]. This is particularly apparent in germ-free animals, in which the immune system is morphologically and functionally underdeveloped [11], [12] and immune maturation can only be triggered by the introduction of intestinal contents or faeces from conventional animals [13]–[15].

The composition of the gut microbiota depends on a multitude of factors that can be either host-dependent or host-independent. The latter includes gestational age, mode of delivery, nutrition, rearing environment, and antibiotic exposure [16]. For example, microbial colonization of the gut in infants delivered by caesarean section is delayed compared to naturally-delivered infants. Strong compositional differences in intestinal microbiota appear to reflect differences in skin, vaginal and faecal microbiota of the mother [17], [18]. Recent work suggests a direct link between specific gut microbiota composition in early life and the subsequent predisposition to a number of important human diseases [19]–[25]. Our previous work has identified strong relationships involving environmental microbiota, antibiotic treatment, gut microbial composition and immune development and function [26]. The present work is a further investigation of the impact of very early microbial colonization on gut mucosal immunity, independent of differences in nutritional and maternal factors and in the context of excessive hygiene. We used piglets that were initially colonized in outdoor (extensive) and indoor (intensive) environments and then transferred and reared in isolators maintained to a high level of hygiene. Detailed microbial diversity composition of outdoor- and isolator-reared animals has been described by Schmidt *et al.*, 2011. In brief, this work revealed that although the initial microbial exposure was identical, the microbial succession and stabilization events observed in naturally-reared outdoor animals [26] did not occur in isolator-reared animals, which maintained a highly diverse microbiota containing a large number of distinct phylotypes. In the current paper, development of the gut in these same animals was assessed by morphological and molecular analysis, with particular emphasis on the expression of genes related to immunity.

## Materials and Methods

### Ethics Statement

All animal studies were performed according to the regulations and guidance provided under the UK Home Office Animals (Scientific Procedures) Act 1986. Experimental protocols were approved under project license number PPL 30/2482.

### Experimental animals and tissue collection

Twelve Large White×Landrace sows (*Sus scrofa*), housed in either an outdoor (extensive) or an indoor (intensive) facility, were artificially inseminated by the same boar to minimize genetic variation among the offspring. Three piglets from each outdoor-housed sow (OUT) and indoor-housed sow (IN) (36 piglets in total) were housed with the sow until weaning at day 28. Three piglets from each outdoor-housed sow and each indoor-housed sow (36 piglets in total) were transferred to individual isolator units at the School of Clinical Veterinary Science (University of Bristol, UK) at 24 h of age (OIs and InIs, respectively). These animals were fed a commercial porcine milk replacer (PiggiMilk, Parnutt Foods Ltd) dispensed by an automated liquid feeding system until weaning on day 28. From day 29 onwards, all piglets were fed creep feed (Multiwean, SCA NUTRITION Ltd) *ad libitum*. The experiment was run in three consecutive replicates, using four sows and 24 piglets in every replicate (Fig. S1).

Six randomly chosen piglets per treatment group were sacrificed by injection of sodium pentobarbitone (Euthesate, Willows Francis Veterinary Ltd) at day 5, 28, and 56. Body weight of the animals was recorded at this time. The ileum, defined as the region corresponding to 75% in length from the pyloric sphincter, was excised. Ileal tissue samples were stored in RNAlater solution (Ambion) for microarray and real-time PCR analyses. Ileal tissue samples were cut into pieces of 2–3 cm and fixed in Carnoy's fixative for histological examination.

### Tissue processing for histological analysis

Ileal tissue of animals from all treatment groups OUT, IN, OIs and InIs was analyzed for gross histology. Ileal tissue samples were fixed for three hours in Carnoy's fixative (60% (v/v) ethanol, 30% (v/v) chloroform and 10% (v/v) glacial acetic acid) at room temperature with constant agitation. The samples were transferred to 70% ethanol and stored at room temperature until embedded in cold-curing resin (orientated for transverse sectioning) using Technovit 8100 (Heraeus Kulzer) according to the manufacturer's instructions. The embedded tissue was mounted onto Histoblocs using Technovit 3040 (Heraeus Kulzer). Four micron sections were cut using a rotary microtome (Leica Autocut) fitted with a glass knife (TAAB Laboratories Equipment Ltd.). Three sections were taken per slide at 100 µm, 200 µm and 300 µm into the tissue, resulting in 9 sections per animal.

Tissue sections were stained using standard Haemotoxylin/Eosin methods, mounted in Histomount and examined with a Zeiss Axioskop microscope equipped with ×10 and ×20 objectives. Images were taken using a QImaging camera and Image Pro Plus software. At least 12 images from each animal were examined in a blinded fashion and gross histological appearance noted for each animal.

### Mucosa-adherent microbiota analysis

For analyses of the mucosa-adherent microbiota, ileal tissue was cut open and contents were removed. The tissue was then washed with ice-cold phosphate buffered saline (PBS) and incubated overnight in ice-cold PBS/0.1% Tween 20 (Sigma-Aldrich) with shaking. Detached bacteria were harvested by centrifugation at 10,000× g for 10 min at 4°C. Total DNA from the pellet was isolated using a DNA Spin Kit for Soil® (QBiogene Inc).

Amplification of the 16S rRNA genes was carried out with the universal primer set 27F (5′-AGAGTTTGATCMTGGCTCAG-3′) and 1492R (5′-ACGGCTACCTTGTTACGACTT-3′) [27]. PCR cycling conditions were: one cycle at 94°C for 5 min, followed by 30 cycles at 94°C for 30 s, 57°C for 30 s, and 72°C for 2 min, with a final extension at 72°C for 10 min. For the second step of the PCR amplification, the primers MF (5′-ATTACCGCGGCTGCTGG-3′) and MR-GC (5′-CCTACGGGAGGCAGCAG-3′ with GC-clamp 5′-CGCCCGCCGCGCGCGGCGGGCGGGGCGGGGGCACGGGGGG-3′) [28] were used with one cycle at 94°C for 5 min, followed by 35 cycles at 94°C for 30 s, 55°C for 30 s, and 72°C for 2 min, with a final extension at 72°C for 10 min. These primers bind the variable V3 region of the 16S rRNA gene. PCR products were visualized on 1.5% agarose gel in TBE buffer stained with ethidium bromide and stored at −20°C. PCR products were separated by DGGE according to the specifications of Muyzer and co-workers [28] using a D-Code Universal Mutation Detection System (Bio-Rad). DGGE banding patterns were analyzed using FPQuest software (BioRad).

Clustering of microbial profiles was performed to visualize hierarchical structure and treatment differences/similarities among the dataset. Comparisons of DGGE pattern profiles were performed using Dice's similarity coefficient (*D*_sc_) analysis. *D*_sc_ values were compared based on presence or absence of bands. Dice's coefficient is defined as *DSC* = 2j/(a+b), where j is the number of common bands between samples A and B; a and b are the total number of bands in samples A and B, respectively. This coefficient ranges from 0 (no common bands) to 100% (identical bands patterns). Similarity was visualized by dendograms using the unweighted pair group method using the arithmetic averages (UPGMA) algorithm [29].

### Microarray hybridizations and data analysis

RNA was isolated from ileal tissue (200 mg) of OIs and InIs animals and processed for hybridization to the GeneChip Porcine Genome Array (Affymetrix) using the One-Cycle Target Labeling Kit (Affymetrix) as described previously [26].

Quality analysis, normalization (gcRMA), statistical analysis, and heatmap generation was performed with the freely available software packages R (http://www.r-project.org) and Bioconductor (http://www.bioconductor.org) [30]. In particular, we used the moderated F-test provided by the Bioconductor package *limma* to test for differential expression [31]. As the biological sample-to-sample variability was very different between the three different time-points but also between the different groups, it was decided not to fit one linear model to all data. Instead, data were restricted to each of the three time-points (day 5, 28 and 56) and then to the comparison OIs vs InIs. A linear model with the factors replicate (three levels) and group (two levels) was fitted and *P*-values for the group effect were calculated with *limma*. Differences in gene expression between the treatment groups were considered significant using *P*<0.01 and −2≤ fold change ≥2 as a cut-off. A heatmap was generated with the R-package *gplots* using a subset of the significantly up- or down-regulated genes. Microarray data were submitted to the National Center for Biotechnology Information (NCBI) Gene Expression Omnibus (accession number GSE15256; http://www.ncbi.nlm.nih.gov/geo).

All differentially expressed genes (*P*<0.05) were imported into the MetaCore™ software suite (GeneGo Inc., St Joseph, MI) to generate pathway maps. Porcine Affymetrix probeset IDs were converted into human Affymetrix probeset IDs using the annotation supplied by Tsai *et al.* [32]. Integrated pathway enrichment analysis was performed using the knowledge-based canonical pathways and endogenous metabolic pathways. Ranking of the relevant integrated pathways was based on *P*-values calculated using hypergeometric distribution.

### Real-time PCR analysis of differentially expressed genes

The significance of differential expression between OIs and InIs uncovered by Affymetrix microarray analyses was further validated using real-time PCR. Two micrograms of total RNA isolated from the ileum (N = 6, isolated for microarray analysis) were reverse transcribed into cDNA using the High Capacity cDNA Reverse Transcription Kit (Applied Biosystems) with random primers. Real-time PCR analysis was performed using a 7500 Fast Real-Time PCR System (Applied Biosystems) with the Power SYBR Green PCR Master Mix (Applied Biosystems) according to the manufacturer's recommendations. Primers for *ACTB*, *G1P2*, *DDX58*, *IFIT1*, *IRF7*, *ZBP1*, *IRP6*, *MX* and *USP18* (Sigma-Aldrich, Table S1) were designed for the porcine sequences of interest using Primer Express Software v3.0 (Applied Biosystems). PCR cycling conditions were: one cycle at 95°C for 10 min, followed by 40 cycles at 95°C for 15s and 60°C for 1 min, ending with a dissociation step. All samples were run in triplicate. *ACTB* was selected as a reference gene for normalization due to its low variation between the samples in the microarray analysis. Data were analyzed by Student's t-test, with *P*<0.05 considered statistically significant.

## Results

### Isolator-rearing significantly reduces growth until weaning

**Figure 1:**
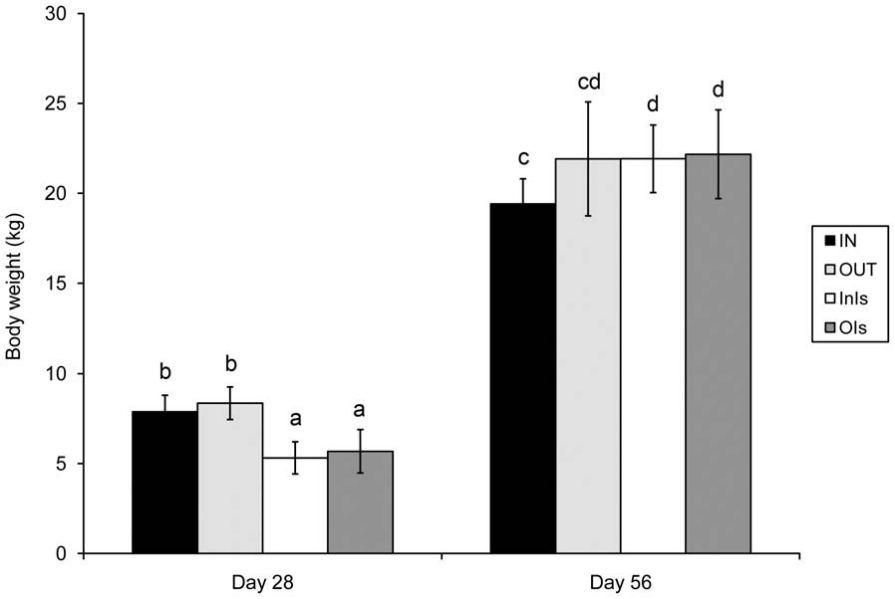
Body weight of sow-reared and isolator-reared animals. Body weight (BW) of all experimental animals at day 28 and 56 is shown. At day 28, a significant reduction in BW was observed for animals housed in isolators compared to sow-reared animals. InIs and OIs animals were significantly heavier than IN animals at day 56.

Body weight (BW) of all experimental animals was recorded at day 28 and 56 (Fig. 1). At day 28, mean BW was 8.36±0.91, 7.89±0.91, 5.68±1.21 and 5.32±0.90 for OUT, IN, OIs and InIs, respectively. A statistically significant reduction of BW was observed in animals housed in isolators compared to sow-reared animals (*P* = 0.0017 for OIs and *P* = 0.0006 for InIs compared to their respective sow-reared littermates). No differences in BW were observed between the IN and OUT animals or InIs animals and OIs animals. At day 56, mean BW was 21.92±3.17, 19.42±1.39, 22.17±2.47 and 21.92±1.88 for OUT, IN, OIs and InIs, respectively. InIs and OIs animals were significantly heavier than IN animals (*P* = 0.0272 and *P* = 0.0447) at this age, while no differences in BW were observed between OUT, InIs and OIs.

### Morphological maturation is delayed in isolator-reared animals

**Figure 2:**
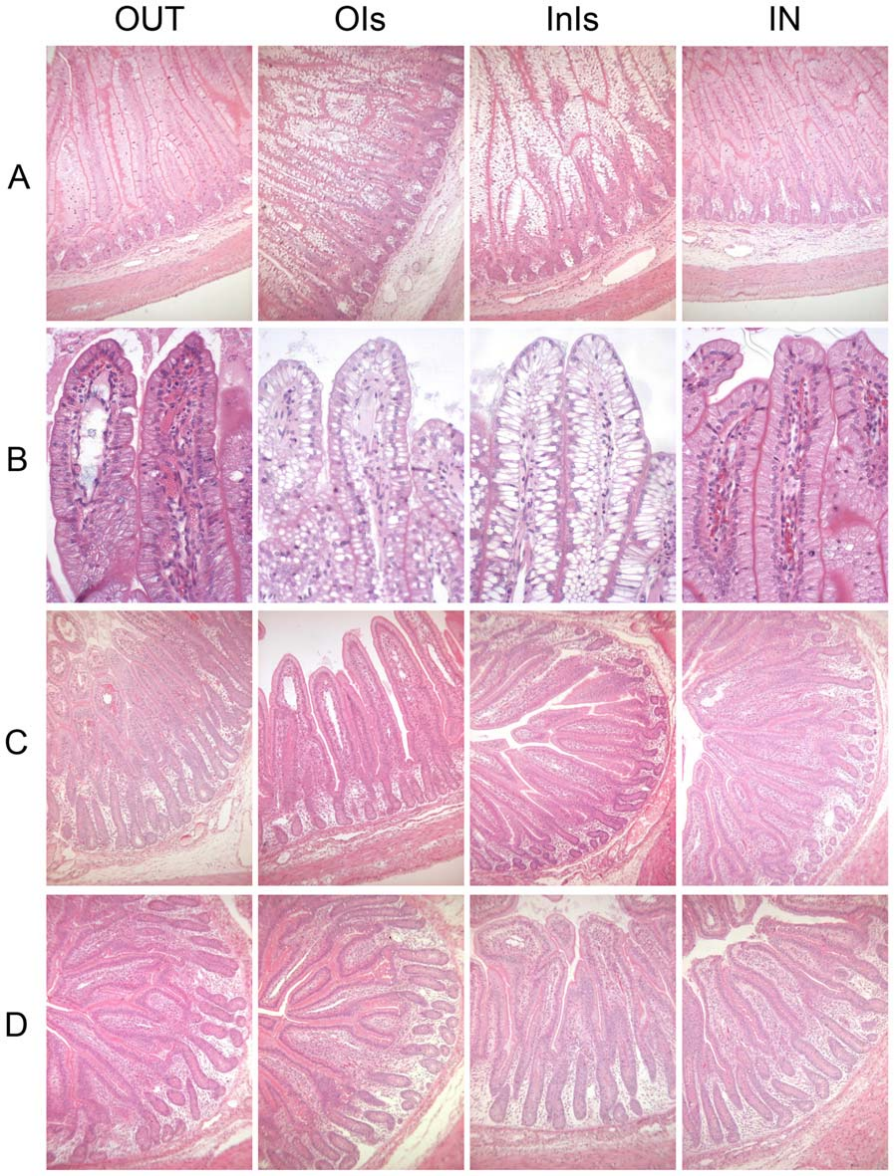
Gut morphology of sow-reared and isolator-reared animals. Representative microscopy images of haematoxylin and eosin–stained cross-sections of tissue taken at 75% of the length of the small intestine from pigs at: (A) day 5 (original magnification 10×), (B) villus tips at day 5 (original magnification 20×), (C) day 28 (original magnification 10×), and (D) day 56 (original magnification 10×).

General morphology of the ileum (defined as the region corresponding to 75% in length from the pyloric sphincter) at day 5 was characterized by long finger-like villi and shallow crypts, with little cellular infiltration into the lamina propria. Ileal sections of IN and OUT piglets looked similar, showing a highly organized epithelial layer with the majority of epithelial nuclei situated basally (Fig. 2A). In contrast, InIs and OIs piglets showed extensive vacuolation in the epithelial cells, particularly at the villus tips. The epithelial layer was less organized, and epithelial nuclei were displaced, sandwiched between two vacuoles, or positioned apically. Vacuoles were present from the crypts to the villus tips. Very little vacuolation was observed in the epithelial cells of OUT animals, with most of the enterocyte nuclei positioned basally. Ileal morphology of the IN animals was comparable to that of the OUT animals. Vascularisation of the epithelium was much more evident in the lamina propria of OUT and IN animals than in OIs and InIs animals (Fig. 2B). Eosin labelling of the brush border was stronger in OUT and IN compared to OIs and InIs.

Weaning is known to be associated with changes in gut morphology. Microscopic examination of the ileum at day 28 (Fig. 2C) showed that crypts were deeper compared to day 5 animals. Villus size and shape was variable, with both short and broad, and longer and narrower villi detected. Lacteals were present in the villus tips of most tissue sections. The epithelial vacuolation observed in OIs and InIs animals at day 5 was no longer apparent at day 28. No gross histological differences between treatment groups were found at this time-point. At day 56 (Fig. 2D), villi were generally short, broad and leaf-like with deep crypts. A large number of intraepithelial lymphocytes were observed in the epithelial layer. Some degree of infiltration of immune cells was visible in most animals. Again, little differences between the treatment groups were observed at this time-point.

### Bacterial diversity of the mucosa-adherent ileal microbiota reflects rearing environment in early life

**Figure 3:**
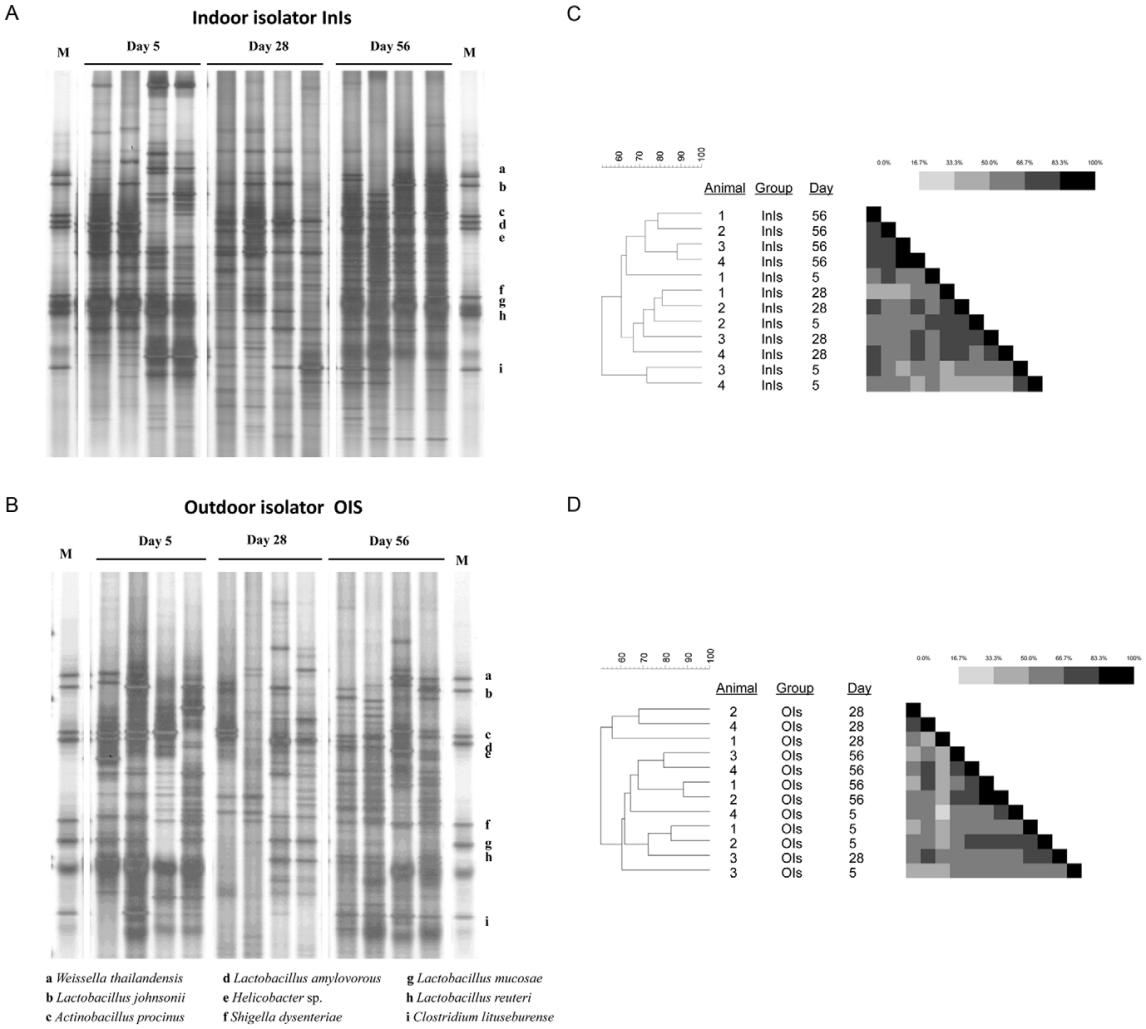
Bacterial diversity of the mucosa-adherent microbiota. DGGE microbial profile at days 5, 28 and 56 in indoor pigs reared in isolators (A) and outdoor pigs reared in isolators (B) at days 5, 28 and 56. The overall DGGE profile showed a highly diverse microbiota at all three sampling time-points, increasing from day 5 towards day 56. Comparisons of DGGE pattern profiles in InIs animals (C) and OIs animals (D) were performed using Dice's similarity coefficient and visualized by dendrograms using the UPGMA algorithm.

Bacterial community profiles of the mucosa-adherent microbiota at the three sampling days were ascertained by DGGE. The overall DGGE profile showed a highly diverse microbiota at all three sampling time-points, increasing from day 5 towards day 56, in both InIs (Fig. 3A) and OIs animals (Fig. 3B). The high diversity of the bacterial profiles within the isolators limited the detection of specific bands. Bands co-migrating with marker bacterial species including *Lactobacillus johnsonii* (b) and *Peptostreptococcus* sp. (i) were identified in most piglets in the InIs group. Bands co-migrating with *L. johnsonii* (b), *Actinobacillus porcinus* (c), and *Peptostreptococcus* sp. (i) were identified in most piglets in the OIs group. Comparisons of DGGE pattern profiles were performed using Dice's similarity coefficient and the similarity was visualized by dendrograms using the UPGMA algorithm. Interestingly, in InIs animals (Fig. 3C), clustering of day 5 and day 28 was separate from day 56 whereas in OIs animals (Fig. 3D), clustering of day 5 and day 56 was observed, indicating that successional events did not occur in this group. This contrasts with the microbial succession and stabilization events reported previously in naturally reared OUT animals [26].

### The ileum transcriptome is mainly affected by microbiota and isolator-rearing, especially in early life

Differences in ileal gene expression between OIs and InIs animals were assessed using the GeneChip Porcine Genome Array. Differential expression between the two treatments was determined using a cut-off of P<0.01 and −2≤ fold change ≥2.

**Figure 4:**
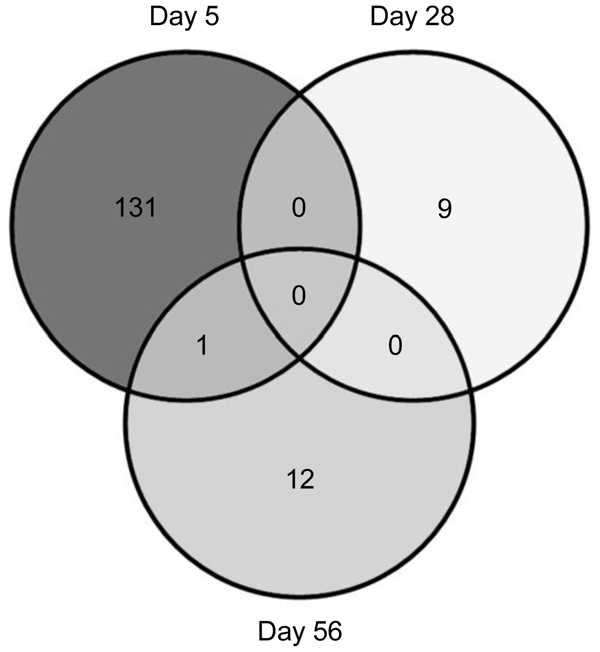
Ileum transcriptome differences between indoor isolator-reared and outdoor isolator-reared animals. Venn diagram of differentially expressed genes at each time-point is shown for the two treatment groups (P<0.01, −2≤ fold change ≥2).

The largest number of significantly changed genes was observed at day 5, with 132 probesets significantly changed between InIs and OIs (Fig. 4 and Table S2). Expression of 93 genes was significantly higher in InIs animals confirming a strong early life environmental effect and reflecting differences in microbial colonization between these treatment groups. These genes included a panel of IFN-induced genes (such as *IFI35*, *IFITM3*, *OAS1*, *ISG20*, *IRF7*, *IFIT3*, *MX1*, *IFIT1*, *IFIT2*, and *G1P2*) and MHC class I genes (*C1orf29*, *CD86*, *HLA-B*, *PSMB9* and *SSA1*). Other immune-related genes included *IL28RA*, *SFTPD*, *OLR1*, *LITAF*, *LGALS9*, *PMCHL1*, *GZMB* and *HSH2D*. Among the 39 transcripts that were higher in OIs animals, immune-related genes included *TCA_HUMAN*, *CXCR4*, *HPGD* and *ARL6IP5*. *ARL6IP5* plays a role in the regulation of cell differentiation, and is upregulated by retinoic acid.

Contrary to these results, differential gene expression at the later time-points was much less pronounced. At day 28, only nine genes were differentially expressed. The expression of *SATB1*, *DDIT4L* and *PAX2* was higher in InIs animals, while that of *GPT2*, *STARD10*, *Q86YJ6*, *H1F0*, *DCXR* and *ITGB4* was higher in OIs animals.

Only 13 transcripts were changed between the two isolator groups at 56d. Twelve of these genes were expressed higher in the OIs group, while only one was higher in the InIs group. The main transcript with increased expression in the OIs group was *ADAMTS18*, which was increased between 16 to 21-fold in outdoor isolator-housed piglets. *ADAMTS18* is implicated in numerous processes such as tumor suppression, cell migration, and immune response [33], [34]. Other highly expressed genes in the OIs group included *BMP2*, *ZNF12*, *MT3* and *ETV1*. *BMP2* belongs to the transforming growth factor-beta (TGFB) superfamily and is involved in the hedgehog pathway, TGF beta signaling pathway, and in cytokine-cytokine receptor interaction.

To further validate these findings, quantitative real-time PCR analysis targeted at various inflammatory mediators and other genes of interest was performed. The selected gene set included *G1P2*, *DDX58*, *IFIT1*, *IRF7*, *ZBP1*, *IRP6*, *MX*, and *USP18*. This analysis correlated well with the Affymetrix microarray results (Table 1). The pattern of up- or down-regulation was consistent between the two platforms, although the magnitude of fold change was generally lower in the RT-PCR approach compared to the Affymetrix microarray analysis.

**Table 1:** Verification of microarray results by real-time PCR.

			Real-time PCR OIs vs InIs			Affymetrix microarray	
Gene	Day	OIs	InIs	Fold change	*P*-value	Fold change	*P*-value
*G1P2*	5	8.74±0.29	4.96±2.62	−13.67	0.01655	−50.69	0.0049
*DDX58*	5	7.80±0.59	5.47±1.77	−5.00	0.02221	−17.33	0.0047
*IFIT1*	5	11.05±0.30	6.88±2.97	−17.99	0.01824	−31.6	0.0006
*IRF7*	5	7.02±0.47	5.79±1.14	−2.34	0.04623	−7.88	0.0057
*ZBP1*	5	9.39±0.83	7.10±1.22	−4.88	0.00447	−13.75	0.0084
*IRP6*	5	9.65±0.96	4.66±2.57	−31.79	0.00369	−26.97	0.0086
*MX*	5	8.64±0.64	5.48±1.66	−8.91	0.00403	−13.35	0.0090
*USP18*	5	14.29±0.90	11.18±2.02	−8.59	0.01115	−11.65	0.0011

The results for the comparison of OIs versus InIs are expressed as mean ΔCt ± SD, N = 6.

### Functional pathway analysis reveals significant activation of innate immune pathways in isolator environments

**Figure 5:**
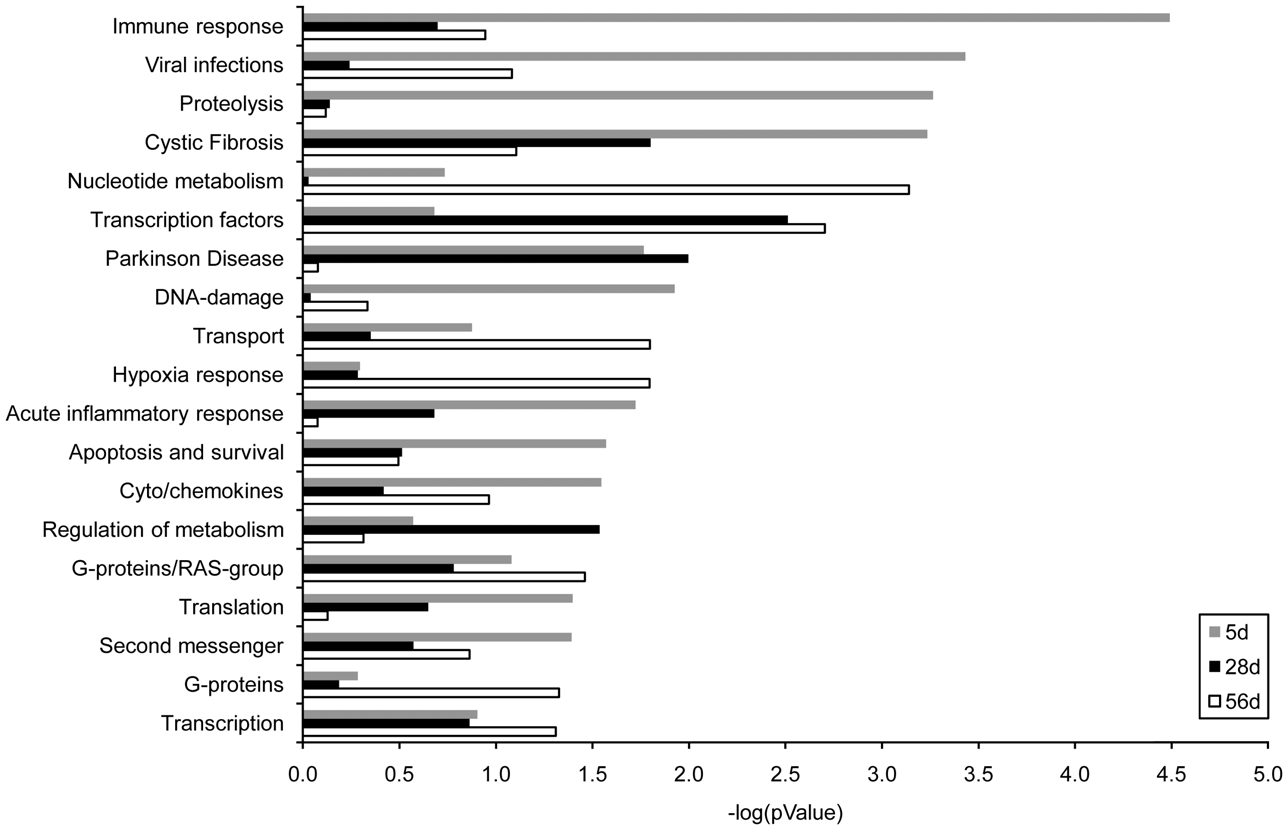
Pathway analysis of genes differentially expressed in indoor isolator-reared and outdoor isolator-reared animals. Differentially expressed genes (P<0.05) were imported into GeneGo MetaCore analytical software to determine significantly enriched canonical pathways in each group. Data represent the distribution in cell process categories of statistically significantly enriched pathways (P<0.05) between the two treatment groups.

Pathway analysis with MetaCore™ software allowed identification of functional categories affected by isolator-rearing. Pathways that were differentially expressed between the housing treatments at day 5 primarily involved the immune response and related functional groups, while at the later time-points a majority of pathways clustered in nucleotide metabolism and transcription factors groups (Fig. 5). More detailed examination of the immune response pathways (Table 2) showed that, in agreement with the downregulation of IFN-induced genes, the *Immune response_IFN alpha/beta signaling pathway* was one of the most affected immune response pathways. At day 5, this pathway was significantly decreased in the OIs group compared to the InIs group (*P* = 0.00133). Eight out of the 24 genes present in this pathway were significantly affected. Similar treatment effects were noted for the *Immune response_IL-22 signaling pathway*, *Immune response_IL-23 signaling pathway* and *Immune response_Th17 cell differentiation*.

**Table 2:** Immune response pathways in animals housed in different environments at day 5, 28 and 56.

Regulatory processes/Immune response	P-Value	Sign.*	Total**
**OIs vs InIs Day 5**		13	34
Immune response_Alternative complement pathway	6.732E-06	11	26
Immune response_Antigen presentation by MHC class I	0.00001	11	34
Immune response_Antiviral actions of Interferons	0.00020	12	43
Immune response_Lectin induced complement pathway	0.00050	12	45
Immune response_Classical complement pathway	0.00078	8	24
Immune response_IFN alpha/beta signaling pathway	0.00123	12	59
Chemotaxis_Leukocyte chemotaxis	0.00883	8	33
Immune response_IL-22 signaling pathway	0.01076	6	22
Immune response_IL-23 signaling pathway	0.01474	7	31
Immune response_MIF - the neuroendocrine-macrophage connector	0.02441	7	31
* Immune response_Th17 cell differentiation	0.02441	9	48
Immune response _Immunological synapse formation	0.03588	5	20
Neurophysiological process_PGE2-induced pain processing	0.03650	8	41
Apoptosis and survival_Lymphotoxin-beta receptor signaling	0.03775	7	34
Immune response_TCR and CD28 co-stimulation in activation of NF-kB	0.03897	6	28
Immune response_HTR2A-induced activation of cPLA2	0.04569	13	34
**OIs vs InIs Day 28**			
Immune response_IL-3 activation and signaling pathway	0.01165	5	30
Immune response_Antigen presentation by MHC class II	0.01260	3	11
Immune response_Role of the Membrane attack complex in cell survival	0.02715	4	25
**OIs vs InIs Day 56**			
Immune response_ICOS pathway in T-helper cell	0.00129	8	37
Immune response_Antigen presentation by MHC class II	0.00303	4	11
Immune response_NFAT in immune response	0.00304	8	42
Immune response_Antigen presentation by MHC class I	0.00372	6	26
Immune response_NF-AT signaling and leukocyte interactions	0.00667	7	38
Immune response _CCR3 signaling in eosinophils	0.00798	9	59
Immune response_TCR and CD28 co-stimulation in activation of NF-kB	0.01453	6	34
Chemotaxis_Lipoxin inhibitory action on fMLP-induced neutrophil chemotaxis	0.01453	6	34
Chemotaxis_Inhibitory action of lipoxins on IL-8- and Leukotriene B4-induced neutrophil migration	0.01668	6	35
Immune response_T cell receptor signaling pathway	0.01676	7	45
Immune response_MIF - the neuroendocrine-macrophage connector	0.03578	5	31
Immune response_Function of MEF2 in T lymphocytes	0.03804	6	42
Immune response_CD28 signaling	0.04207	6	43
Immune response_Regulation of T cell function by CTLA-4	0.04536	5	33

Differentially expressed genes (P<0.05) were imported into GeneGo MetaCore analytical software to determine the significantly enriched immune response pathways.

*The number of genes on each map that are differentially expressed in the specific treatment comparison.

**The total number of genes on each map.

Comparative transcriptome patterns of InIs versus OIs at day 5 were most congruous with the disease profile of ‘nut hypersensitivity’. The differentially expressed set included pathways such as *Chemotaxis_Leukocyte chemotaxis*, *Immune response_Immunological synapse formation*, *Immune response_IL-22 signaling pathway*, *Immune response_ MIF the neuroendocrine-macrophage connector*, and *Immune response_TCR and CD28 co-stimulation in activation of NF-kB*.

The *Immune response_Antigen presentation by MHC class I* pathway was similarly affected by isolator-rearing. The OIs group displayed differential expression of 11genes of the pathway compared to the InIs animals (*P* = 0.00001). At day 56, expression of genes in this pathway was significantly decreased in OIs animals compared to InIs animals (*P* = 0.00372). Interestingly, the pathway for *Immune response_Antigen presentation by MHC class II* was enriched at day 28 (*P* = 0.01260) and 56 (*P* = 0.00303) in InIs animals compared to OIs animals, although the differences were significant only for a small number of genes.

**Figure 6:**
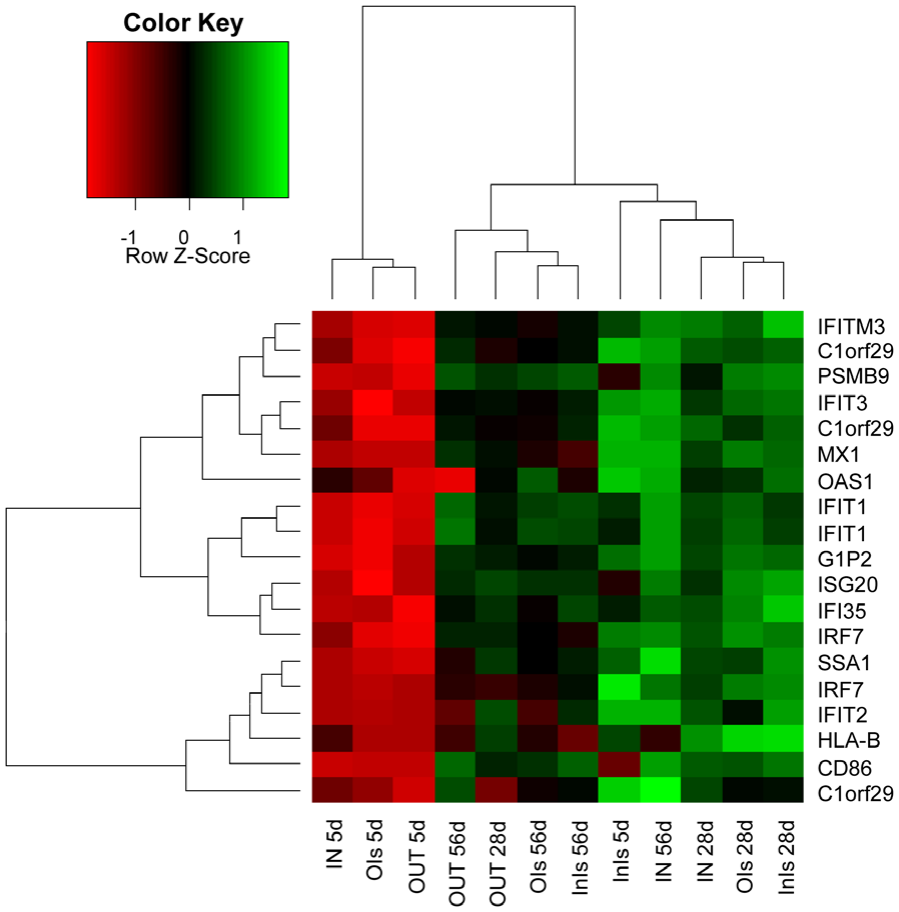
Heatmap of IFN-induced gene subset. A heatmap of IFN-related genes was generated from a subset extracted from the list of significantly expressed genes. Columns represent individual arrays, while rows represent specific genes of interest. The Z-score depicts a measure of distance, in standard deviations, away from the mean. The relative value for each gene is depicted by color intensity, with green indicating upregulated and red indicating downregulated genes.

To investigate further the influence of the rearing environment on IFN-induced genes, a heatmap was generated for this subset of differentially expressed genes (Fig. 6). Gene information from the OUT and IN groups was included in this comparison since the differential expression of similar genes in these treatment groups has been demonstrated in our previous work [26]. The resulting heatmap showed that at day 5 the IN, OIs and OUT groups are clustered together and separated from the InIs group, illustrating the specific upregulation of IFN-induced genes in the InIs group. OUT animals at day 28 and 56 still showed relatively low expression of these genes, while the OIs animals clustered with InIs animals at these time-points. The IN animals also displayed higher expression of the corresponding genes at day 28 and 56. The heatmap analysis suggests therefore that the reduced expression of IFN-regulated genes associated with the initial outdoor environment was lost during isolator rearing.

## Discussion

The postnatal period is a ‘critical window’ for development of the immune system. The current study assessed firstly, how immediate early-life microbial exposure influences morphological and molecular development of the pig gut and secondly, how microbial restriction during development influences immune function in the longer-term. Piglets were obtained from either an extensive, outdoor farming facility (OUT) or an intensive, indoor facility (IN). All piglets remained on the sow during the first day of life to enable ingestion of colostrum as well as the acquisition of maternal and environmental microbiota. Animals were then transferred to individual isolator units (OIs and InIs) to allow the study of ‘early-acquired’ microbiota on immune development. The overall health performance of animals in the isolators was good although body weight tended to be lower than that of the sow-reared animals, despite the *ad libitum* access to milk. Interestingly, after weaning and the switch to a solid diet, InIs animals were significantly heavier than IN animals which had been sow-reared.

A marked effect of isolator-rearing, but not of the birth environment, on closure of the neonatal gut was noted. Interestingly, although a similar delay in gut closure has been observed in young germfree pigs and in animals mono-associated with *Lactobacillus fermentum*, *Escherichia coli* or *Staphylococcus epidermidis* [35], [36], the animals in the current study were colonized by large numbers of bacteria, inferring a more important role for sow's milk and suckling in functional gut closure, rather than microbial colonization per se. This is consistent with previous published data [37].

The paper by Schmidt *et al* (2011) shows that the mucosa-adherent microbiota of the isolator-reared animals remained very diverse. Our previous work on sow-reared indoor and outdoor animals revealed that microbial diversity of outdoor-born piglets decreased as the pigs progressed developmentally from neonate to adult-like [26]. This suggests that strong environmental and immune-related selective pressures drive events which shape the microbiota, but importantly, this stabilization process requires continual microbial exposure throughout development. In this study, the isolation of piglets shortly after birth restricted subsequent environmental microbial exposure. Consequently, the bacterial subsets colonizing the gut throughout the experiment were mainly acquired during the first days of life. Microbial expansion and high diversity was seen in both isolator groups after weaning, and suggests that succession and stabilization of the mucosa-adherent microbiota was significantly impaired by isolation of these animals.

Affymetrix microarray analysis of the transcriptome differences between the treatment groups revealed strong differential gene expression between the indoor isolator-reared and outdoor isolator-reared animals at day 5 after birth. InIs piglets had increased gene expression levels of the IFN alpha/beta signaling pathway as well as IFN-induced genes at this time-point. Type I IFNs have many biological functions, including innate, cellular and humoral adaptive immune responses [38], [39]. The MHC class I complex was similarly affected by isolator-rearing. The activation of Type I IFN genes and MHC class I in InIs concurs with our previous work [26], and shows that environmental differences affecting gut microbiota composition dramatically influence the level of immune activation in the very early stages of life. This was further supported by the increased activation of innate immune genes such as *CCL28* (chemotactic for resting CD4^+^ and CD8^+^ T cells and eosinophils) and *PHF11* (regulator of Th1 cytokine gene expression) and immune response pathways associated with IL-22, IL-23 and Type I IFN in InIs animals. The consequences of elevated innate immune activation in early life are not fully appreciated but may alter immunologic programming and predispose to a variety of immune-mediated diseases including allergy and autoimmunity [40]. In support of this, recent data shows that excessive hygiene interferes with the normal homeostatic processes of immune regulation mediated by CD4^+^FoxP3^+^ cells (Lewis *et al.*, submitted).

The effects of the birth environment on IFN-signaling were abrogated at later time-points and indeed, the overall differences in gene expression were very low between the treatment groups. This data concurs with other work by our group (Schmidt *et al.*, 2011) showing that the microbiota profile of the isolator-reared groups exhibited high microbial diversity with little differences between these groups at the adult life stage. Hence, the absence of differential IFN gene expression at later time-points corresponds with the convergence of microbiota composition between the two isolator groups. This has important implications regarding continuous microbial exposure during development and suggests that sustained microbial exposure is required to maintain any early-life benefits derived from microbial colonization.

The composition of the gut microbiota is clearly important to immune function as previously shown; work on microbiota analysis of outdoor sow-reared animals and indoor sow-reared animals revealed large compositional differences which corresponded to major differences in immune activation [26]. This data strongly infers that the succession events that lead to a stable adult microbiota depend not just on early acquisition of microbes, but also on continuous exposure to a natural, highly diverse environmental microbiota throughout development. Conversely, reduced microbial exposure as a result of excessive hygiene appears to interfere with the normal processes of microbiota succession and stabilization [12] and alters immune development. Published data on migrant children, in which the microbiota is not fully mature, has revealed an increased susceptibility to immune diseases following relocation to high risk urban areas, a phenomenon not observed in parents [41]–[43]. This data supports the notion that environment, and in particular microbial exposure throughout early life, is an important risk factor in the development of immune diseases in children.

## Supporting Information

Figure S1 **Experimental design of the animal study.**
(TIF)

Table S1 **PCR primer sequences.** Porcine gene-specific primers were designed using Primer Express Software v3.0.
(DOCX)

Table S2 **Treatment-dependent differential gene expression at all three time-points.** Differentially expressed genes at each time point are shown for the comparison of InIs versus OIs (*P*<0.01, −2≤ fold change ≥2, *N* = 6).
(DOCX)
