# Supplementary figures and images for: Restricting Microbial Exposure in Early Life Negates the Immune Benefits Associated with Gut Colonization in Environments of High Microbial Diversity
Source: PLoS One. 2011 Dec 22;6(12):e28279. doi: 10.1371/journal.pone.0028279 (PMC3245219; doi:10.1371/journal.pone.0028279)

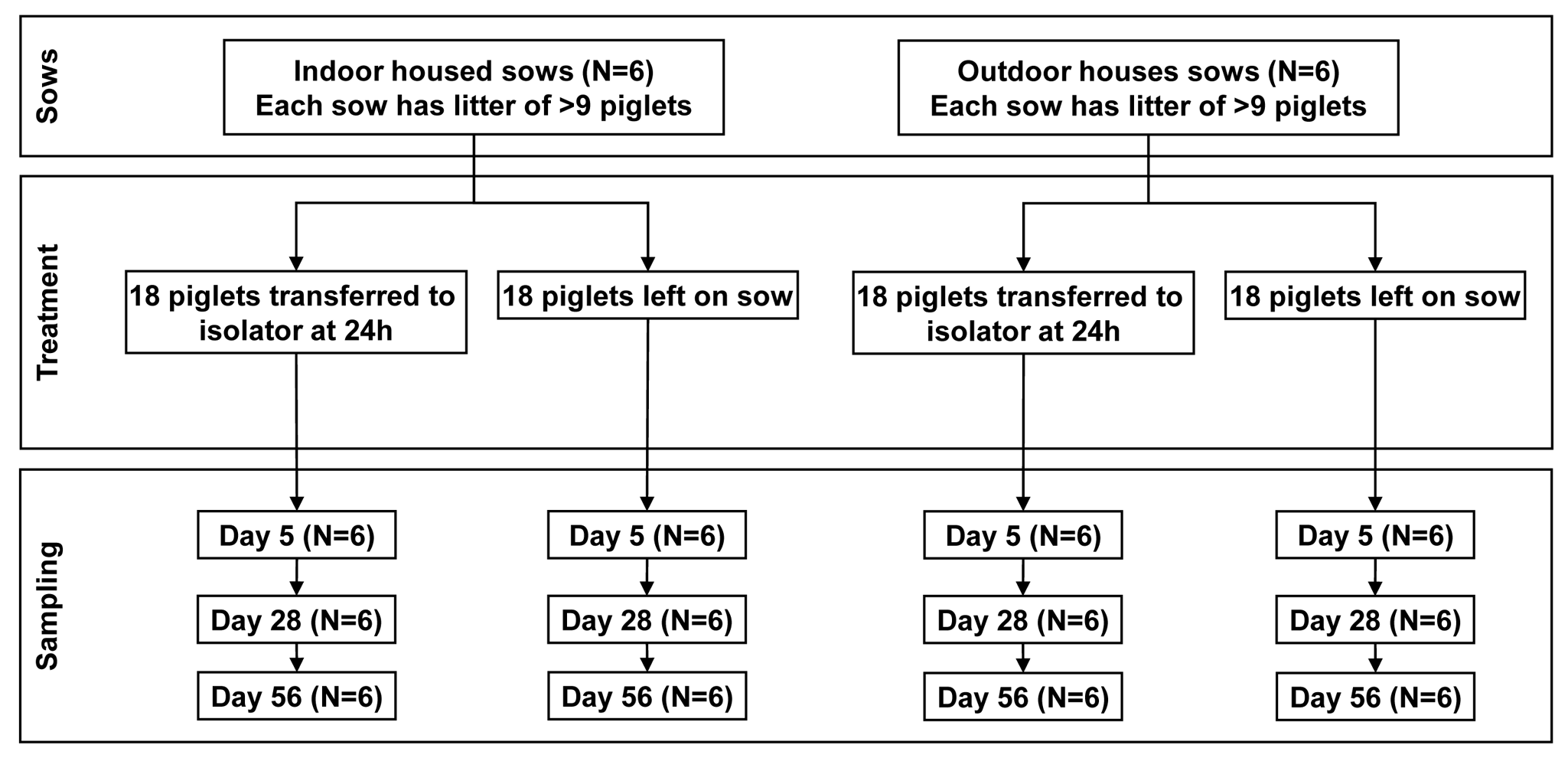

Supplement: Figure S1 — Experimental design of the animal study. (TIF) [file pone.0028279.s001.tif]
