# Supplementary material for: Restricting Microbial Exposure in Early Life Negates the Immune Benefits Associated with Gut Colonization in Environments of High Microbial Diversity
Source: PLoS One. 2011 Dec 22;6(12):e28279. doi: 10.1371/journal.pone.0028279 (PMC3245219; doi:10.1371/journal.pone.0028279)
Supplement: Table S1 — PCR primer sequences. Porcine gene-specific primers were designed using Primer Express Software v3.0. (DOCX) [file pone.0028279.s002.docx]

**Table S1.** **PCR primer sequences.**

| **Gene** | **Forward Primer** | **Reverse Primer** |
| --- | --- | --- |
| *ACTB* | CTCTTCCAGCCCTCCTTCCT | CGACGTCGCACTTCATGATG |
| *DDX58* | GGCTGCCACATCCATTGG | CAAAAGCAGCAAAGAGGACAAA |
| *G1P2* | TGCTGGGAGGCAAGGAGAT | GTCCGATGCCATCATGCA |
| *IFIT1* | GGGTATGCAATCACCATCTATCG | AAAATGGCCCATCGAAACCT |
| *IRF7* | CTGCGATGGCTGGATGAAG | TAAAGATGCGCGAGTCGGA |
| *IRP6* | CAATCGCTTCAATGTGGAAGAAG | CCCTCAATTAAGAGGCATTGGA |
| *MX* | GGCGTGGGAATCAGTCATG | AGGAAGGTCTATGAGGGTCAGATCT |
| *USP18* | CCCCTTCCAGCTGCTCTTG | CATTGGTATATTGTACTTCTGGAGACAGT |
| *ZBP1* | GCTCTTTCCTGCTGGCATTG | AGGCAGGCGGTTAGTTTTCA |

Porcine gene-specific primers were designed using Primer Express Software v3.0.
