# Supplementary material for: Restricting Microbial Exposure in Early Life Negates the Immune Benefits Associated with Gut Colonization in Environments of High Microbial Diversity
Source: PLoS One. 2011 Dec 22;6(12):e28279. doi: 10.1371/journal.pone.0028279 (PMC3245219; doi:10.1371/journal.pone.0028279)
Supplement: Table S2 — Treatment-dependent differential gene expression at all three time-points. Differentially expressed genes at each time point are shown for the comparison of InIs versus OIs (P<0.01, −2≤ fold change ≥2, N = 6). (DOCX) [file pone.0028279.s003.docx]

**Table S2A - Transcripts differentially expressed between the OIs and InIs treatment groups at day 5 (P<0.01, -2≤FC≥2**). Positive fold-change indicates increase in OIs/decrease in InIs; negative fold-change indicates increase in InIs/decrease in OIs.

| **affy.id** | **Gene Name** | **Product** | **FC** | **p-value** |
| --- | --- | --- | --- | --- |
| Ssc.12.1.S1_at | SULT1E1 | Estrogen sulfotransferase (Sulfotransferase, estrogen- preferring) (EST-1) | 10.05 | 0.0019 |
| Ssc.428.6.S1_a_at | TCA_HUMAN | T-cell receptor alpha chain C region | 4.17 | 0.0091 |
| Ssc.7176.1.A1_at | CXCR4 | C-X-C chemokine receptor type 4 (CXC-R4) (CXCR-4) (Stromal cell- derived factor 1 receptor) (SDF-1 receptor) (Fusin) (Leukocyte-derived seven transmembrane domain receptor) (LESTR) (LCR1) (FB22) (NPYRL) (HM89) (CD184 antigen) | 3.51 | 0.0047 |
| Ssc.15335.1.S1_at | C6orf32 |  | 3.39 | 0.0073 |
| Ssc.18388.1.A1_at | COPG | Coatomer gamma subunit (Gamma-coat protein) (Gamma-COP) | 3.16 | 0.0039 |
| Ssc.1732.1.S1_at | ARL6IP5 | ADP-ribosylation-like factor 6 interacting protein 5; glutamate transporter EEAC1-associated protein; dermal papilla derived protein 11; putative MAPK activating protein PM27; PRA1 domain family 3; cytoskeleton related vitamin A responsive protein | 3.11 | 0.0055 |
| Ssc.21796.1.S1_at | SORL1 | Sortilin-related receptor precursor (Sorting protein-related receptor containing LDLR class A repeats) (SorLA) (SorLA-1) (Low-density lipoprotein receptor relative with 11 ligand-binding repeats) (LDLR relative with 11 ligand-binding repeats) (LR11). | 3.07 | 0.0044 |
| Ssc.22678.1.S1_at | SMARCAL1 | SWI/SNF-related matrix-associated actin-dependent regulator of chromatin a-like 1; HepA-related protein; SMARCA-like protein 1 | 2.99 | 0.0015 |
| Ssc.11174.1.S1_at | SLC26A2 | Sulfate transporter (Diastrophic dysplasia protein) | 2.81 | 0.0073 |
| Ssc.4808.1.S1_at | Q8NEX2 |  | 2.59 | 0.0006 |
| Ssc.26932.2.A1_at | Q96IS5 |  | 2.55 | 0.0038 |
| Ssc.3966.1.S1_at | Q9ULS3 |  | 2.44 | 0.0030 |
| Ssc.6104.1.A1_at | Q6UXI0 |  | 2.37 | 0.0004 |
| Ssc.6028.1.S1_at | Q8TAD7 |  | 2.26 | 0.0070 |
| Ssc.31059.1.S1_at | TLE1 | Transducin-like enhancer protein 1 (ESG1) | 2.25 | 0.0007 |
| Ssc.15266.1.S1_at | HPGD | 15-hydroxyprostaglandin dehydrogenase [NAD+] (PGDH) | 2.25 | 0.0011 |
| Ssc.10650.1.A1_at | Q8N8X8 |  | 2.25 | 0.0032 |
| Ssc.7242.1.A1_at | STAU2 | Staufen homolog 2; staufen (Drosophila, RNA-binding protein) 2; staufen (Drosophila, RNA-binding protein) homolog 2 | 2.23 | 0.0046 |
| Ssc.22413.1.A1_at | TPBG | 5T4 oncofetal trophoblast glycoprotein; 5T4-antigen | 2.20 | 0.0059 |
| Ssc.4805.1.S1_at | RPS27 | 40S ribosomal protein S27 (Metallopan-stimulin 1) (MPS-1) | 2.20 | 0.0026 |
| Ssc.29365.1.A1_at | FRK | Tyrosine-protein kinase FRK (Nuclear tyrosine protein kinase RAK) | 2.19 | 0.0067 |
| Ssc.1271.3.S1_at | NP_057160 | Protein CGI-96 (PNAS-4). | 2.17 | 0.0027 |
| Ssc.5008.1.A1_at | GSTA4 | Glutathione S-transferase A4-4 (GST class-alpha) | 2.16 | 0.0089 |
| Ssc.20917.1.S1_at | C5orf13 | Neuronal protein 3.1 (p311 protein) | 2.13 | 0.0000 |
| Ssc.7893.2.A1_at | MTSS1 | Metastasis suppressor protein 1 (Missing in metastasis protein) (Metastasis suppressor YGL-1) | 2.13 | 0.0037 |
| Ssc.1271.1.S1_at | NP_057160 | Protein CGI-96 (PNAS-4) | 2.11 | 0.0091 |
| Ssc.19338.1.A1_at | NP_056337 | Dynein 2 light intermediate chain isoform 1 | 2.11 | 0.0008 |
| Ssc.8258.1.A1_at | RPL24 | 60S ribosomal protein L24 (L30) | 2.10 | 0.0048 |
| Ssc.27502.1.S1_at | KCNH5 | Potassium voltage-gated channel subfamily H member 5 (Voltage-gated potassium channel subunit Kv10.2) (Ether-a-go-go potassium channel 2) (hEAG2) | 2.09 | 0.0013 |
| Ssc.1056.1.A1_at | PRKAR2B | cAMP-dependent protein kinase type II-beta regulatory chain | 2.08 | 0.0041 |
| Ssc.7810.1.A1_at | CXXC4 | CXXC finger 4; Dvl-binding protein IDAX; inhibition of the Dvl and Axin complex | 2.07 | 0.0010 |
| Ssc.7039.1.A1_at | NGFRAP1 | p75NTR-associated cell death executor (Nerve growth factor receptor associated protein 1) (Ovarian granulosa cell 13.0 kDa protein HGR74) | 2.07 | 0.0011 |
| Ssc.2368.1.S1_at | FLI1 | Friend leukemia integration 1 transcription factor (Fli-1 proto- oncogene) (ERGB transcription factor) | 2.06 | 0.0011 |
| Ssc.14324.1.A1_at | XPR1 | Xenotropic and polytropic retrovirus receptor | 2.06 | 0.0090 |
| Ssc.24128.1.A1_at | O75458 | 20 kDa protein | 2.06 | 0.0030 |
| Ssc.28297.1.A1_at | TMED8 | Transmembrane emp24 domain containing 8 | 2.03 | 0.0084 |
| Ssc.30916.1.A1_at | NP_862830 | Amphoterin induced gene 2; transmembrane protein AMIGO2; alivin 1; differentially expressed in gastric adenocarcinoma | 2.03 | 0.0009 |
| Ssc.10642.1.S1_at | CTNNA3 | Catenin, alpha 3; alpha-catenin-like protein; alpha-T-catenin | 2.02 | 0.0088 |
| Ssc.1584.1.A1_at | MEF2D | Myocyte-specific enhancer factor 2D | 2.01 | 0.0067 |
| Ssc.30869.2.S1_at | C17orf27 |  | -2.02 | 0.0047 |
| Ssc.3411.1.A1_at | ZFP36 | Tristetraproline (TTP) (TIS11A protein) (TIS11) (Zinc finger protein 36 homolog) (Zfp-36) (Growth factor-inducible nuclear protein NUP475) (G0/G1 switch regulatory protein 24) | -2.04 | 0.0086 |
| Ssc.2475.1.S1_at | FIBCD1 | Fibrinogen C domain containing 1 | -2.04 | 0.0025 |
| Ssc.31035.1.S1_at | PSD | Pleckstrin and Sec7 domain containing; pleckstrin and Sec7 domain protein | -2.06 | 0.0086 |
| Ssc.5540.1.S1_at | IFI35 | Interferon-induced 35 kDa protein (IFP 35) | -2.10 | 0.0006 |
| Ssc.2219.1.S1_at | PFKFB4 | 6-phosphofructo-2-kinase/fructose-2,6-biphosphatase 4 (6PF-2-K/Fru- 2,6-P2ASE testis-type isozyme) [Includes: 6-phosphofructo-2-kinase; Fructose-2,6-bisphosphatase | -2.12 | 0.0049 |
| Ssc.24893.1.S1_at | IL28RA | Interleukin 28 receptor, alpha isoform 1; interferon lambda, receptor 1; interleukin or cytokine receptor 2; interleukin 28 receptor A; class II cytokine receptor CRF2/12 | -2.15 | 0.0090 |
| Ssc.27928.1.S1_at | OGFR | Opioid growth factor receptor (OGFr) (Zeta-type opioid receptor) (7-60 protein) | -2.20 | 0.0029 |
| Ssc.23808.2.S1_a_at | CLIC4 | Chloride intracellular channel protein 4 (Intracellular chloride ion channel protein p64H1) | -2.21 | 0.0044 |
| Ssc.11351.1.S1_at | ATP7B | Copper-transporting ATPase 2 (Copper pump 2) (Wilson disease-associated protein) | -2.22 | 0.0094 |
| Ssc.20734.1.S1_at | TRIM26 | Tripartite motif-containing protein 26 (Zinc finger protein 173) (Acid finger protein) (AFP) | -2.35 | 0.0034 |
| Ssc.1295.1.A1_at | CDH4 | Cadherin-4 precursor (Retinal-cadherin) (R-cadherin) (R-CAD) | -2.36 | 0.0013 |
| Ssc.15888.1.S1_at | OLR1 | Oxidised low density lipoprotein (lectin-like) receptor 1; scavenger receptor class E, member 1; lectin-type oxidized LDL receptor 1 | -2.38 | 0.0092 |
| Ssc.3522.2.S1_at | CNP | 2',3'-cyclic-nucleotide 3'-phosphodiesterase (EC 3.1.4.37) (CNP) (CNPase) | -2.38 | 0.0050 |
| Ssc.16160.1.S1_at | CD86 | T lymphocyte activation antigen CD86 precursor (Activation B7-2 antigen) (CTLA-4 counter-receptor B7.2) (B70) (FUN-1) (BU63) | -2.41 | 0.0059 |
| Ssc.14415.1.A1_at | BBAP_HUMAN | B-lymphoma- and BAL-associated protein (Rhysin 2) (Rhysin2) | -2.42 | 0.0071 |
| Ssc.25815.1.A1_at | PARP3 | Poly [ADP-ribose] polymerase-3 (PARP-3) (NAD(+) ADP- ribosyltransferase-3) (Poly[ADP-ribose] synthetase-3) (pADPRT-3) (hPARP-3) (IRT1) | -2.46 | 0.0099 |
| Ssc.23929.1.A1_at | PMCHL1 | Pro-MCH variant (Fragment) | -2.48 | 0.0093 |
| Ssc.30737.1.A1_s_at | PNPT1 | Polyribonucleotide nucleotidyltransferase 1; polynucleotide phosphorylase-like; 3'-5' RNA exonuclease | -2.52 | 0.0071 |
| Ssc.9327.1.A1_at | HSH2D | Hematopoietic SH2 domain containing; adaptor in lymphocytes of unknown function X; hematopoietic SH2 protein | -2.61 | 0.0092 |
| Ssc.7224.1.A1_at | O15050 | PREDICTED: KIAA0342 gene product | -2.65 | 0.0025 |
| Ssc.17424.1.S1_at | C10orf11 | Protein C10orf11 (CDA017) | -2.65 | 0.0069 |
| Ssc.27383.1.A1_at | BCDO2 | Beta,beta-carotene 9',10'-dioxygenase (Beta-carotene dioxygenase 2) (B-diox-II) | -2.67 | 0.0083 |
| Ssc.12758.1.A1_at | PSMB9 | Proteasome subunit beta type 9 precursor (Proteasome chain 7) (Macropain chain 7) (Multicatalytic endopeptidase complex chain 7) (RING12 protein) (Low molecular mass protein 2) | -2.67 | 0.0035 |
| Ssc.18715.1.A1_at | CITED4 | Cbp/p300-interacting transactivator, with Glu/Asp-rich carboxy-terminal domain, 4; transcriptional co-activator 4 | -2.75 | 0.0042 |
| Ssc.27573.1.S1_at |  | 21 kDa protein | -2.76 | 0.0081 |
| Ssc.13226.1.A1_at | BAL_HUMAN | B aggressive lymphoma protein | -2.84 | 0.0083 |
| Ssc.9733.1.S1_at | ZCWCC3 | Zinc finger CW-type coiled-coil domain protein 3 | -2.84 | 0.0055 |
| Ssc.26227.1.S1_at | SETX_HUMAN | Probable helicase senataxin (SEN1 homolog) | -2.89 | 0.0043 |
| Ssc.26119.1.S1_at | HYAL1 | Hyaluronoglucosaminidase 1 isoform 1; hyaluronidase 1; tumor suppressor LUCA-1; plasma hyaluronidase | -2.91 | 0.0025 |
| Ssc.12829.1.A1_at | TRIM4 | Tripartite motif protein 4 | -2.92 | 0.0060 |
| Ssc.21339.1.S1_at | SSA1 | 52 kDa Ro protein (Sjogren syndrome type A antigen) (SS-A) (Ro(SS-A)) (52 kDa ribonucleoprotein autoantigen Ro/SS-A) | -2.93 | 0.0070 |
| Ssc.14340.3.S1_at | LITAF | Lipopolysaccharide-induced tumor necrosis factor-alpha factor (LPS- induced TNF-alpha factor) (P53-induced protein 7) | -2.99 | 0.0078 |
| Ssc.7274.1.A1_at | PRKR | Interferon-induced, double-stranded RNA-activated protein kinase (Interferon-inducible RNA-dependent protein kinase) (p68 kinase) (P1/eIF-2A protein kinase) | -3.02 | 0.0001 |
| Ssc.23500.1.S1_s_at | SFTPD | Pulmonary surfactant-associated protein D precursor (SP-D) (PSP-D) | -3.15 | 0.0055 |
| Ssc.5030.1.A1_at | TRIM22 | Tripartite motif protein 22 (RING finger protein 94) (50 kDa stimulated trans-acting factor) (Staf-50) | -3.20 | 0.0026 |
| Ssc.11098.1.S1_at | IFITM3 | Interferon-induced transmembrane protein 3 (Interferon-inducible protein 1-8U) | -3.41 | 0.0002 |
| Ssc.5887.1.A1_at | SLC37A2 | Solute carrier family 37 (glycerol-3-phosphate transporter), member 2 | -3.46 | 0.0025 |
| Ssc.10593.2.A1_at | C1orf29 | Histocompatibility 28 | -3.47 | 0.0032 |
| Ssc.1031.1.S1_at | OAS1 | 2'-5'-oligoadenylate synthetase 1 ((2-5')oligo(A) synthetase 1) (2-5A synthetase 1) (p46/p42 OAS) (E18/E16). | -3.48 | 0.0019 |
| Ssc.12720.1.S1_at | TM4SF11 | Plasmolipin | -3.48 | 0.0059 |
| Ssc.30169.1.A1_at | C7orf6 |  | -3.53 | 0.0054 |
| Ssc.2641.1.S1_at | UBE2L6 | Ubiquitin-conjugating enzyme E2-18 kDa UbcH8 (Ubiquitin- protein ligase) (Ubiquitin carrier protein) (Retinoic acid induced gene B protein) (RIG-B) | -3.56 | 0.0037 |
| Ssc.4939.1.A1_at | GZMB | Granzyme B precursor (T-cell serine protease 1-3E) (Cytotoxic T-lymphocyte proteinase 2) (Lymphocyte protease) (SECT) (Granzyme 2) (Cathepsin G-like 1) (CTSGL1) (CTLA-1) (Fragmentin 2) (Human lymphocyte protein) (HLP) (C11) | -3.68 | 0.0086 |
| Ssc.2393.1.S1_at | LCAT | Phosphatidylcholine-sterol acyltransferase precursor (Lecithin-cholesterol acyltransferase) (Phospholipid-cholesterol acyltransferase) | -3.89 | 0.0064 |
| Ssc.1625.1.S1_at | RNF31 | Transcriptional regulator ISGF3 gamma subunit (IFN-alpha responsive transcription factor subunit) (Interferon stimulated gene factor 3 gamma) (ISGF3 p48 subunit) (ISGF-3 gamma) | -3.92 | 0.0005 |
| Ssc.10588.1.A1_at | C1orf29 | Histocompatibility 28 | -3.95 | 0.0004 |
| Ssc.6850.1.A1_at | NANS | Sialic acid synthase (N-acetylneuraminate synthase) (N- acetylneuraminic acid synthase) (N-acetylneuraminate-9-phosphate synthase) (EC 2.5.1.57) (N-acetylneuraminic acid phosphate synthase) | -3.95 | 0.0003 |
| Ssc.6430.1.A1_at | VAPB | Vesicle-associated membrane protein-associated protein B/C (VAMP- associated protein B/C) (VAMP-B/VAMP-C) (VAP-B/VAP-C) | -4.03 | 0.0002 |
| Ssc.28988.1.S1_at | CNP | 2',3'-cyclic-nucleotide 3'-phosphodiesterase (EC 3.1.4.37) (CNP) (CNPase) | -4.10 | 0.0060 |
| Ssc.12286.2.A1_at | PML | Probable transcription factor PML (Tripartite motif protein 19) | -4.12 | 0.0057 |
| Ssc.12286.1.A1_at | PML | Probable transcription factor PML (Tripartite motif protein 19) | -4.13 | 0.0091 |
| Ssc.4617.1.S1_at | CYP2D6 | Cytochrome P450 2D6 (CYPIID6) (P450-DB1) (Debrisoquine 4-hydroxylase) | -4.32 | 0.0002 |
| Ssc.6191.1.S1_at | BBAP_HUMAN | B-lymphoma- and BAL-associated protein (Rhysin 2) (Rhysin2) | -4.35 | 0.0022 |
| Ssc.5104.1.S1_at | NLN1_HUMAN | Nuclear protein 1 (Protein p8) (Candidate of metastasis 1) | -4.56 | 0.0100 |
| Ssc.30869.1.S1_at | C17orf27 |  | -4.60 | 0.0021 |
| SscAffx.1.1.S1_at | ISG20 | Interferon stimulated gene 20kDa; interferon stimulated gene (20kD) | -4.74 | 0.0031 |
| Ssc.13068.1.A1_at | ZC3HDC1 | Zinc finger CCCH type domain containing protein 1 | -4.82 | 0.0004 |
| Ssc.16227.1.S1_at | ZDHHC9 | Zinc finger DHHC domain containing protein 9 (Zinc finger protein 379) (CGI-89) | -4.89 | 0.0012 |
| Ssc.10593.1.S1_at | C1orf29 | Histocompatibility 28 | -5.01 | 0.0013 |
| Ssc.18554.1.S1_x_at | HLA-B | HLA class I histocompatibility antigen, B-7 alpha chain precursor (MHC class I antigen B*7) | -5.11 | 0.0024 |
| Ssc.319.1.S1_at | SFTPD | Pulmonary surfactant-associated protein D precursor (SP-D) (PSP-D) | -5.33 | 0.0006 |
| Ssc.26292.1.S1_at | HIST1H2AC | Histone H2A.l (H2A/l) | -5.36 | 0.0022 |
| Ssc.12504.1.A1_at | FAM14A | Protein FAM14A precursor (TLH29 protein) (pIFI27-like protein) | -5.72 | 0.0046 |
| Ssc.28988.2.S1_at | CNP | 2',3'-cyclic-nucleotide 3'-phosphodiesterase (EC 3.1.4.37) (CNP) (CNPase) | -5.82 | 0.0064 |
| Ssc.10993.1.S1_a_at | LGALS9 | Galectin-9 (HOM-HD-21) (Ecalectin) | -5.95 | 0.0006 |
| Ssc.4425.1.S1_at | PCDH15 | Protocadherin 15 precursor | -6.26 | 0.0074 |
| Ssc.25739.1.S1_at | IRF7 | Interferon regulatory factor 7 (IRF-7) | -6.31 | 0.0090 |
| Ssc.6433.1.S1_at | Q9P2E3 |  | -6.42 | 0.0004 |
| Ssc.21162.1.S1_s_at | IRF7 | Interferon regulatory factor 7 (IRF-7) | -7.88 | 0.0057 |
| AFFX-Ss_IRP_5_at |  |  | -8.61 | 0.0047 |
| Ssc.6433.2.S1_at | Q9P2E3 |  | -9.07 | 0.0014 |
| Ssc.30724.1.S1_at | HERC6 | Hect domain and RLD 6 | -9.08 | 0.0006 |
| Ssc.17861.1.A1_at | MEIS1 | Homeobox protein Meis1 | -9.41 | 0.0012 |
| Ssc.26189.1.S1_a_at |  | 28 kDa protein | -10.78 | 0.0011 |
| Ssc.31140.1.S1_at | IFIT3 | Interferon-induced protein with tetratricopeptide repeats 3 (IFIT-3) (IFIT-4) (Interferon-induced 60 kDa protein) (IFI-60K) (ISG-60) (CIG49) (Retinoic acid-induced gene G protein) (RIG-G) | -11.48 | 0.0031 |
| Ssc.336.1.S1_at | USP18 | Ubl carboxyl-terminal hydrolase 18 (Ubl thiolesterase 18) (ISG15-specific processing protease) (43 kDa ISG15-specific protease) (hUBP43) | -11.65 | 0.0008 |
| Ssc.7558.1.A1_at | RAB3C | Ras-related protein Rab-3C | -11.86 | 0.0000 |
| Ssc.20101.1.S1_at | G1P3 | Interferon-induced protein 6-16 precursor (Ifi-6-16) | -12.51 | 0.0004 |
| Ssc.221.1.S1_at | MX1 | Interferon-regulated resistance GTP-binding protein MxA (Interferon- induced protein p78) (IFI-78K) | -13.35 | 0.0004 |
| Ssc.30532.1.A1_at | XRCC2 | DNA-repair protein XRCC2 (X-ray repair cross-complementing protein 2) | -13.69 | 0.0041 |
| Ssc.26005.1.S1_at | ZBP1 | Z-DNA binding protein 1 (Tumor stroma and activated macrophage protei DLM-1) | -13.75 | 0.0010 |
| Ssc.13128.1.A1_at | Q8IY21 |  | -14.51 | 0.0001 |
| Ssc.22620.1.S1_at | IFIT2 | Interferon-induced protein with tetratricopeptide repeats 2 (IFIT-2) (Interferon-induced 54 kDa protein) (IFI-54K) (ISG-54 K) | -14.99 | 0.0027 |
| Ssc.15885.1.S1_at | DDX58 | DEAD/H (Asp-Glu-Ala-Asp/His) box polypeptide RIG-I; RNA helicase; DEAD/H (Asp-Glu-Ala-Asp/His) box polypeptide | -15.57 | 0.0016 |
| Ssc.21.1.S1_s_at | DDX58 | DEAD/H (Asp-Glu-Ala-Asp/His) box polypeptide RIG-I; RNA helicase; DEAD/H (Asp-Glu-Ala-Asp/His) box polypeptide | -17.33 | 0.0014 |
| Ssc.30752.2.A1_at | IFIT1 | Interferon-induced protein with tetratricopeptide repeats 1 (IFIT-1) (Interferon-induced 56 kDa protein) (IFI-56K) | -24.83 | 0.0016 |
| Ssc.6172.1.S1_at | NP_954590 | XIAP associated factor-1 isoform 1 | -25.55 | 0.0008 |
| AFFX-Ss_IRP_M_at |  |  | -26.97 | 0.0015 |
| Ssc.30752.1.S1_at | IFIT1 | Interferon-induced protein with tetratricopeptide repeats 1 (IFIT-1) (Interferon-induced 56 kDa protein) (IFI-56K) | -31.60 | 0.0015 |
| Ssc.11557.1.A1_at | G1P2 | Ubiquitin cross-reactive protein precursor (Interferon-induced 17 kDa protein) (Interferon-induced 15 kDa protein) | -50.69 | 0.0001 |
| AFFX-Ss_IRP_3_at |  |  | -52.69 | 0.0002 |
| Ssc.286.1.S1_s_at | NP_542388 | Viperin; similar to inflammatory response protein 6 | -60.94 | 0.0001 |

**Table S2B - Transcripts differentially expressed between the OIs and InIs treatment groups at day 28 (P<0.01, -2≤FC≥2**). Positive fold-change indicates increase in OIs/decrease in InIs; negative fold-change indicates increase in InIs/decrease in OIs.

| **affy.id** | **Gene Name** | **Product** | **FC** | **P-value** |
| --- | --- | --- | --- | --- |
| Ssc.7458.1.A1_at | GPT2 | Alanine aminotransferase 2; glutamic-pyruvate transaminase 2 | 5.11 | 0.0078 |
| Ssc.17934.1.S1_at | STARD10 | PCTP-like protein (PCTP-L) (StAR-related lipid transfer protein 10) (StARD10) (START domain-containing protein 10) (CGI-52) (Serologically defined colon cancer antigen 28) (Antigen NY-CO-28) | 3.12 | 0.0049 |
| Ssc.4665.1.S1_at | Q86YJ6 |  | 2.24 | 0.0077 |
| Ssc.18486.2.S1_at | H1F0 | Histone H1.0 (H1(0)) (Histone H1') | 2.14 | 0.0072 |
| Ssc.23192.2.S1_a_at | DCXR | Dicarbonyl/L-xylulose reductase; carbonyl reductase; kidney dicarbonyl reductase; carbonyl reductase II | 2.05 | 0.0015 |
| Ssc.8924.1.A1_at | ITGB4 | Integrin beta-4 precursor (GP150) (CD104 antigen) | 2.05 | 0.0002 |
| Ssc.23516.1.S1_at | SATB1 | DNA-binding protein SATB1 (Special AT-rich sequence binding protein 1) | -2.07 | 0.0018 |
| Ssc.11437.1.A1_at | DDIT4L | DNA-damage-inducible transcript 4-like; regulated in development and DNA damage response 2 | -2.10 | 0.0033 |
| Ssc.21096.1.S1_at | PAX2 | Paired box protein Pax-2 | -3.15 | 0.0044 |

**Table S2C - Transcripts differentially expressed between the OIs and InIs treatment groups at day 56 (P<0.01, -2≤FC≥2**). Positive fold-change indicates increase in OIs/decrease in InIs; negative fold-change indicates increase in InIs/decrease in OIs.

| **affy.id** | **Gene Name** | **Product** | **FC** | **P-value** |
| --- | --- | --- | --- | --- |
| Ssc.28905.2.S1_at | ADAMTS18 | ADAMTS-18 precursor (A disintegrin and metalloproteinase with thrombospondin motifs 18) (ADAM-TS 18) (ADAM-TS18) | 21.15 | 0.0081 |
| Ssc.28905.1.A1_at | ADAMTS18 | ADAMTS-18 precursor (A disintegrin and metalloproteinase with thrombospondin motifs 18) (ADAM-TS 18) (ADAM-TS18) | 16.53 | 0.0080 |
| Ssc.21290.1.S1_at | Q6PI50 |  | 6.09 | 0.0022 |
| Ssc.12544.1.S1_at | Q6IA24 |  | 2.53 | 0.0078 |
| Ssc.29004.1.S1_at | BMP2 | Bone morphogenetic protein 2 precursor (BMP-2) (BMP-2A) | 2.52 | 0.0035 |
| Ssc.10665.1.A1_at | ZNF12 | RB-associated KRAB repressor | 2.38 | 0.0078 |
| Ssc.19607.1.S1_at | Q68DL8 |  | 2.36 | 0.0024 |
| Ssc.15694.1.S1_at | MT3 | Metallothionein-III (MT-III) (Growth inhibitory factor) (GIF) (GIFB) | 2.35 | 0.0056 |
| Ssc.5022.1.A1_at | NP_114162 | NYD-SP14 protein | 2.11 | 0.0035 |
| Ssc.7186.1.A1_at | ETV1 | Ets translocation variant 1 (ER81 protein) | 2.06 | 0.0010 |
| Ssc.4190.1.S1_at | BMP2 | Bone morphogenetic protein 2 precursor (BMP-2) (BMP-2A) | 2.05 | 0.0028 |
| Ssc.19005.1.A1_at | Q96CC2 | SEMACAP3 protein | 2.01 | 0.0031 |
| Ssc.26189.1.S1_a_at |  | 28 kDa protein | -2.33 | 0.0059 |
